# PSC-derived intestinal organoids with apical-out orientation as a tool to study nutrient uptake, drug absorption and metabolism

**DOI:** 10.3389/fmolb.2023.1102209

**Published:** 2023-01-18

**Authors:** Panagiota Kakni, Carmen López-Iglesias, Roman Truckenmüller, Pamela Habibović, Stefan Giselbrecht

**Affiliations:** ^1^ MERLN Institute for Technology-Inspired Regenerative Medicine, Department of Instructive Biomaterials Engineering, Maastricht University, Maastricht, Netherlands; ^2^ Microscopy CORE lab, Maastricht Multimodal Molecular Imaging Institute (M4I), Maastricht University, Maastricht, Netherlands

**Keywords:** intestinal organoids, epithelial polarity, drug discovery, nutrient uptake, barrier integrity, gastrointestinal model

## Abstract

Intestinal organoids recapitulate many features of the *in vivo* gastrointestinal tract and have revolutionized *in vitro* studies of intestinal function and disease. However, the restricted accessibility of the apical surface of the organoids facing the central lumen (apical-in) limits studies related to nutrient uptake and drug absorption and metabolism. Here, we demonstrate that pluripotent stem cell (PSC)-derived intestinal organoids with reversed epithelial polarity (apical-out) can successfully recapitulate tissue-specific functions. In particular, these apical-out organoids show strong epithelial barrier formation with all the major junctional complexes, nutrient transport and active lipid metabolism. Furthermore, the organoids express drug-metabolizing enzymes and relevant apical and basolateral transporters. The scalable and robust generation of functional, apical-out intestinal organoids lays the foundation for a completely new range of organoid-based high-throughput/high-content *in vitro* applications in the fields of nutrition, metabolism and drug discovery.

## 1 Introduction

The intestinal epithelium is a highly organized, dynamic cell layer that creates a tight barrier between the luminal contents and the rest of the body. It is responsible for food digestion, nutrient transport and protection of the body from infections. Most of the orally administered drugs are also absorbed by the intestine and undergo first-pass metabolism. The establishment and maintenance of epithelial cell polarity, with distinct apical and basolateral surfaces, are pivotal for the proper conduction of these functions (Schneeberger et al., 2018). The apical side faces the intestinal lumen and mediates nutrient uptake and interactions with microorganisms. The basolateral side faces the basement membrane and surrounding tissues and is responsible for nutrient transport to the bloodstream and intercellular communication. Disruption of polarity has been associated with cancer (Fatehullah et al., 2013), microvillus inclusion disease, malnutrition, fetal diarrheal disorder and inflammatory bowel disease (Schneeberger et al., 2018). In the past years, animal models and cancer cell lines were considered the gold standard for modeling the functions of the intestine, but they do not accurately represent the human situation *in vivo* (Negoro et al., 2018; Youhanna and Lauschke, 2021).

Intestinal organoids are three-dimensional (3D) structures that recapitulate multiple features of the *in vivo* intestine: they contain all the major cell types (e.g., Lgr5^+^ stem cells, Goblet cells, Paneth cells, enterocytes etc.) of the *in vivo* intestine (Sato et al., 2009; Spence et al., 2011), organize into crypt-villus structures and possess tissue polarity, thus mimicking the architecture and function of the intestinal epithelium. Intestinal organoids have been utilized in numerous studies related to nutrient transport, metabolism and drug development (Zietek et al., 2015; Zietek et al., 2020; Negoro et al., 2018; Onozato et al., 2018; Janssen et al., 2020; Kasendra et al., 2020) and thus hold a great promise as a tool to study intestinal development, physiology and disorders (Günther et al., 2019; Kakni et al., 2022b). Pluripotent stem cells (PSC) have shown a remarkable ability to differentiate towards all the cell types of the body and they can be used as a source for generating intestinal organoids (Spence et al., 2011). PSC-derived organoids have great potential for patient-specific disease modeling and high-throughput drug screenings (Negoro et al., 2018; Frum and Spence, 2020; Heydari et al., 2021; Turhan et al., 2021; Xia et al., 2021; Günther et al., 2022; Hu and Lazar, 2022). They have been found to express fundamental drug-metabolizing enzymes, and uptake and efflux transporters (Onozato et al., 2018; Janssen et al., 2020; Zietek et al., 2020; Sasaki et al., 2021). However, the limited access to the apical surface of the organoid’s epithelium hampers the assessment of intestinal permeability and drug or nutrient absorption. In addition, the presence of viscous gels surrounding the organoids, such as Matrigel, can further limit or slow down the diffusion of drugs or other compounds into the central lumen of the organoids. To overcome these, numerous studies have utilized organoids as cell sources to create 2D monolayers, e.g., on porous membranes of culture inserts. Even though this approach grants access to both the apical and basal surfaces, the intricate 3D structure of the organoids is largely lost. Recently, intestinal organoids with apical-out orientation were established using both adult (Co et al., 2019; Co et al., 2021; Li et al., 2020; Nash et al., 2021) and pluripotent stem cells (Kakni et al., 2022a). These innovative organoid models maintain the 3D architecture and function, and at the same time provide direct access to the apical side of the epithelium.

In this study, we utilized apical-out intestinal organoids, derived from human PSCs and we demonstrate their functionality with regard to nutrient uptake, drug transport and metabolism. Similar to a previously described method (Kakni et al., 2022a), organoids were cultured in a suspension system following a stepwise differentiation method. After maturation, they expressed all the major cell types found in the *in vivo* intestine and they demonstrated strong epithelial barrier integrity, nutrient uptake, lipid metabolic functions and the presence of transporters, which are important drug and nutrient targets. Thus, these apical-out human intestinal organoids are a valuable *in vitro* model for future studies of nutrient transport, drug development and pharmacokinetics.

## 2 Materials and methods

A full list of materials is provided in the Supplementary Table S1.

### 2.1 Maintenance of PSCs

The human embryonic stem cell (hESC) line WA09 (H9) was obtained from WiCell. The ES cell line was maintained in feeder-free conditions using mTESR1 (StemCell Technologies), and passaged onto Matrigel (Corning)-coated tissue culture dishes every 4–5 days (Ludwig et al., 2006). For passaging, H9 cells were treated with Gentle Cell Dissociation Reagent (StemCell Technologies) for 6 min and then lifted from the plate using sterile cell scrapers (VWR). The split ratio was 1:50. The passage number of the cells used in this study was between 50–60.

### 2.2 Fabrication and preparation of microwell arrays

Microthermoforming was used for the fabrication of microwells as described previously (Giselbrecht et al., 2006; Kakni et al., 2020). Briefly, 50 μm thick polymer films were used to form microwells, which were 500 μm wide and 300 μm deep. Each array contained 289 U-bottom microwells. For sterilization, microwells were treated stepwise with decreasing concentrations of 2-propanol (VWR) (100%–70%–50%–25%–10%) and then washed twice with Dulbecco’s phosphate buffered saline (PBS; Sigma-Aldrich). The incubation time for every step of the sterilization process was 30 s. When placed in 24-well plates, elastomeric O-rings (ERIKS) were mounted on top of the microwell arrays in order to keep them in place.

### 2.3 Differentiation of PSCs towards intestinal organoids

The protocol for directed differentiation of intestinal organoids was carried out as previously described (Kakni et al., 2022a). hESC colonies were dissociated into single cells using TrypLE^™^ Express Enzyme (Thermofisher) and seeded on microwell arrays at a density of 1,000 cells/microwell in mTesR1 supplemented with Y-27632 (10 μΜ; Tocris) to create embryoid bodies (EBs). For definitive endoderm (DE) differentiation, EBs were treated with Activin A (100 ng/mL; Cell Guidance Systems) in RPMI 1640 (Thermofisher) medium supplemented with increasing concentrations (0%, 0.2%, 2%) of HyClone defined fetal bovine serum (dFBS; Thermofisher) for 3 days. The following 4 days, the DE spheroids were incubated with Fibroblast Growth Factor 4 (FGF4) (500 ng/mL; R&D Systems) and CHIR99021 (3 μM; Stemgent) to further differentiate towards hindgut. Full medium exchange (500 μL/well) was performed daily. Thereby, to maintain the spheroids in the microwells, the plate was slightly tilted and the medium was aspirated from the sidewalls.

To promote intestinal differentiation, two different approaches were taken. Specifically, for organoids with the apical side in (facing the lumen), the hindgut spheroids were collected, embedded in 50 μL Matrigel and plated as droplets (Matrigel domes) into tissue culture–treated 24-well plates. Matrigel was allowed to polymerize at 37°C and 5% CO_2_ for 15 min and afterwards, the Matrigel drops containing the spheroids were overlaid with Advanced Dulbecco’s Modified Eagle Medium/Ham’s F-12 (DMEM/F-12) supplemented with B27 (1 X), N2 (1 X), Hepes (15 mM), penicillin/streptomycin (1 X), L-glutamine (1 X) (all Thermofisher), Epidermal Growth Factor (EGF) (50 ng/mL; R&D systems), Noggin (100 ng/mL; R&D systems) and R-Spondin (500 ng/mL; R&D systems). The medium (700 μL/well) was refreshed every 4 days. After reaching full maturation (day 39), organoids were used for downstream experiments.

For organoids with the apical side out (facing the culture medium), the hindgut spheroids were collected on day 8 and placed in suspension culture in non-tissue culture-treated 6-well plates. To avoid surface-cell adherence, the plates were coated with 1% Pluronic F-108 (Sigma-Aldrich) solution in PBS for 2 h at 37°C and then washed two times with PBS. The medium (1.5 mL/well) had the same composition as the apical-in organoids, but in this case, 2% Matrigel was added as a supplement. After reaching full maturation (day 39), organoids were used for downstream experiments.

### 2.4 Epithelial barrier integrity

The permeability of fluorescence markers into the lumen of organoids was tested using 4 kDa fluorescein isothiocyanate (FITC)-labeled dextran (Sigma-Aldrich). Intact organoids were pelleted and resuspended in growth medium containing 2 mg/mL of FITC-dextran for 30 min at room temperature (RT). As a control, organoids were disrupted with 2 mM ethylenediamine tetraacetic acid (EDTA; VWR) in Hanks’ balanced salt solution (no calcium and no magnesium; Thermofisher) on ice for 15 min and then resuspended in FITC-dextran solution. Finally, organoids were washed and mounted and immediately imaged using confocal laser scanning microscopy (Leica TCS SP8).

### 2.5 Fatty acid absorption assay

Apical-in (domes) organoids were treated with 5 mM EDTA in PBS for 1 h on a shaking platform at 4°C to remove the Matrigel. All organoids (apical-in and apical-out) were washed with DMEM (Thermofisher) with no phenol red and incubated in a solution containing 5 μM fluorescent fatty acid analog C1-BODIPY-C12 (Thermofisher) and 5 μM fatty-acid-free bovine serum albumin (BSA; Sigma-Aldrich) for 30 min at 37°C. Afterwards, organoids were fixed in 4% formaldehyde (VWR) in PBS for 30 min. Finally, samples were stained with phalloidin and counterstained with 4′, 6-diamidino-2-phenylindole (DAPI) (both Sigma-Aldrich), and imaged with confocal laser scanning microscopy (Leica TCS SP8). For every independent experiment (4 in total), 10 organoids were analyzed.

### 2.6 Drug treatments

Apical-in and apical-out intestinal organoids were treated with 20 μM rifampicin (Sigma-Aldrich) and 100 nM 1, 25-dihydroxyvitamin D3 (Sigma-Aldrich), which are known inducers of the cytochrome CYP3A4 and the apical transporter multidrug resistance mutation 1 (MDR1), for 48 h. In addition, organoids were treated with 100 μM verapamil (Sigma-Aldrich) and 20 μM ivacaftor (Selleck Chemicals), which are known inhibitors of CYP3A4 and MDR1, for 48 h. As controls, organoids were treated with 0.1% dimethyl sulfoxide (DMSO; VWR). Following treatment, organoids were harvested for RNA isolation.

### 2.7 RNA isolation and quantitative real-time PCR (qPCR)

Organoids were collected and the total RNA was extracted using the RNeasy Mini Kit (Qiagen) as per manufacturer instructions. cDNA was synthesized using the iScript cDNA Synthesis Kit (Bio-Rad). qPCR was performed on a CFX96 real-time PCR detection system (Bio-Rad) using the iQ SYBR Green Supermix (Bio-Rad). Gene expression for each sample was normalized using the hypoxanthine phosphoribosyltransferase (HPRT) housekeeping gene. Data analysis was performed according to the 2^−ΔΔCT^ method. The results represent the mean values of three independent experiments (*n* = 3). The primer sequences are listed in the supplementary material (Supplementary Table S2).

### 2.8 Transmission electron microscopy (TEM)

Initially, organoids underwent a chemical fixation for 3 h at RT with 1.5% glutaraldehyde (Merck) in 0.067 M cacodylate (Acros Organics) buffered to pH 7.4% and 1% sucrose (Merck). Later on, they were washed with 0.1 M cacodylate buffer and postfixed with 1% osmium tetroxide (Agar Scientific) in the same buffer containing 1.5% potassium ferricyanide (Merck) for 1 h in the dark at 4°C. After rinsing with Mill-Q water, organoids were dehydrated at RT in a graded ethanol (VWR) series (70%, 90%, up to 100%), infiltrated with Epon, embedded in the same resin and polymerized for 48 h at 60°C. Using a diamond knife (Diatome), ultrathin sections (60 nm) were cut on a Leica UC7 ultramicrotome and transferred onto 50 mesh copper grids covered with formvar and carbon film. Sections were then stained with 2% uranyl acetate in 50% ethanol and lead citrate. Finally, the sections were imaged in a Tecnai T12 electron microscope equipped with an Eagle 4 k × 4 k CCD camera (Thermofisher) or Veleta 2 k × 2 k CCD camera (Olympus Soft Imaging).

### 2.10 Nile red staining

For lipid droplets visualization, the organoids were fixed in 4% formaldehyde in PBS for 30 min and then incubated with 500 nmol/L Nile red (Sigma-Aldrich) for 15 min at RT. Then, organoids were washed twice with PBS, counterstained with the nuclear dye DAPI and imaged with a confocal laser scanning microscope (Leica TCS SP8).

### 2.11 Immunofluorescence and confocal microscopy

Intestinal organoids were fixed in 4% formaldehyde in PBS for 30 min and then washed with PBS. For permeabilization, organoids were treated with 0.5% Triton X-100 (Sigma-Aldrich) in PBS for 30 min at RT. For blocking, 5% donkey serum in the permeabilization solution was used. Incubation of primary antibodies (full list in Supplementary Table S3) was performed overnight at 4°C and the next day, secondary antibodies (Supplementary Materials) were added for 2 h at RT. Finally, the samples were counterstained with DAPI. For the imaging of the immunostained samples, confocal laser scanning microscopy (Leica TCS SP8) was utilized, and the images were processed with ImageJ. Quantification was performed using the open access software QuPath v0.3.2.

### 2.12 Statistical analysis

Statistical analysis was performed using GraphPad Prism 9 software. Student’s two-tailed *t*-test with Welch’s correction and two-way ANOVA were used to determine statistical significance. Significant differences were defined as *p* < 0.05. *p* values of statistical significance are represented as ****p < 0.0001, ***p < 0.001, **p < 0.01, and *p < 0.05. Error bars in figures indicate standard error of the mean (S.E.M.).

## 3 Results

### 3.1 Apical-out intestinal organoids demonstrate epithelial barrier integrity

**FIGURE 1:**
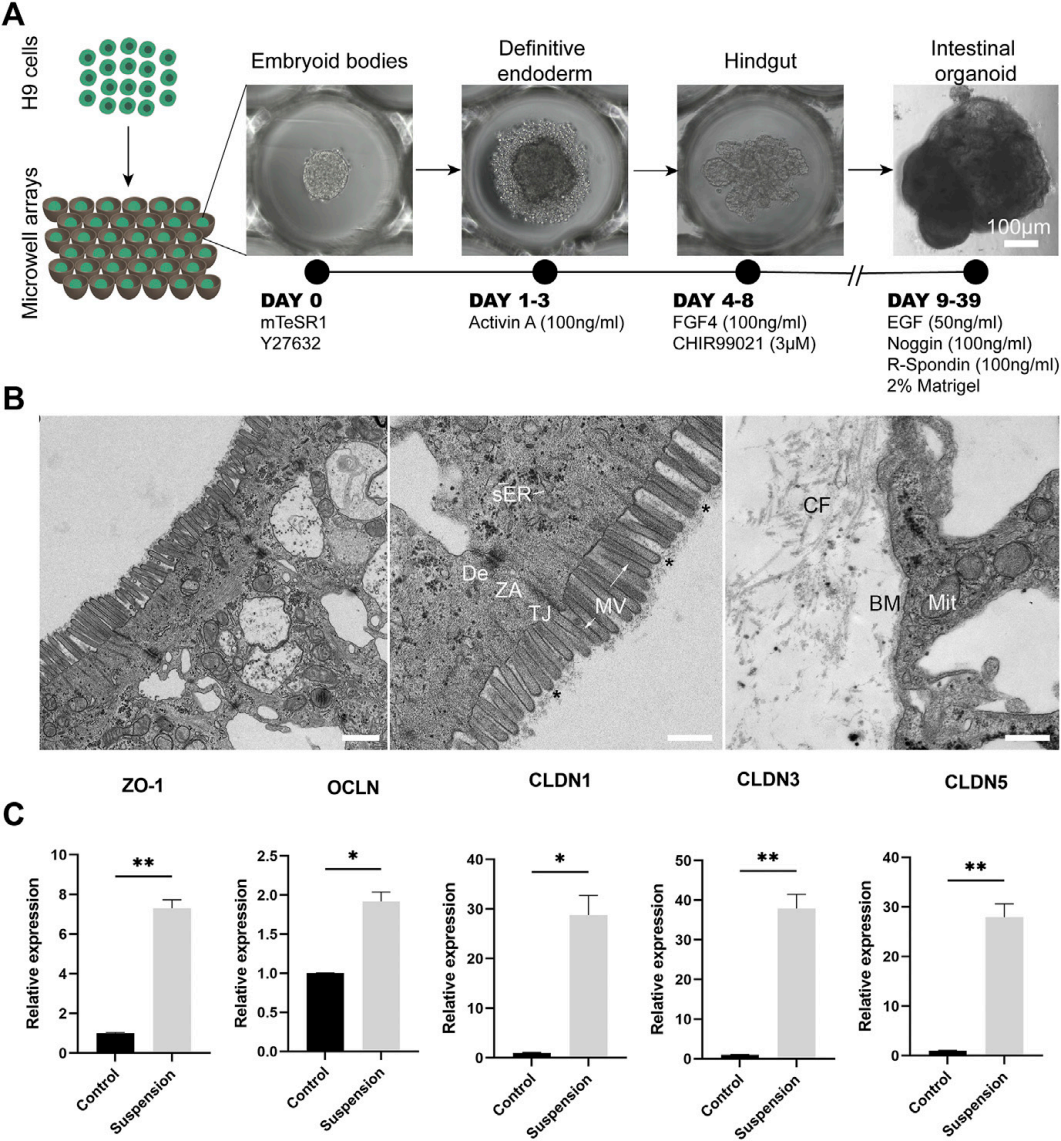
Apical-out intestinal organoids show tight barrier formation. **(A)** Overview of the protocol for the directed differentiation of H9 embryonic stem cells towards intestinal organoids with apical-out orientation. Scale bar: 100 μm. **(B)** On the left, a low magnification TEM image of the epithelial sheet of apical-out organoids demonstrating some enterocytes. In the middle image, TEM indicates the presence of apical microvilli (MV) on the outer surface of organoids. Functional ultrastructural features of the intestinal epithelium are indicated in the apical region of the enterocytes: microvilli (arrows) with core actin filaments and glycocalix (stars), vesicles of smooth endoplasmic reticulum (sER) and intercellular junctions: tight junction (TJ), zonula adherens (ZA) and desmosome (De). In the basal region (right image), the indicated structures correspond to the basement membrane (BM), collagen fibers (CF) and the mitochondria (Mit). Scale bars: 1 μm (left) and 500 nm (middle and right). **(C)** qRT-PCR analysis shows significantly higher expression of the “leak pathway” regulators *ZO-1* and *OCLN* and the “pore pathway” regulators *CLDN1, CLDN3, CLDN5* in the apical-out intestinal organoids compared to undifferentiated control stem cells. Error bars indicate mean ± S.E.M. (*n* = 3).

Culture of organoids in suspension with partial or complete removal of basement membrane (-like) matrix has led to the generation of organoids with reversed polarity, where the apical side is facing outwards to the surrounding culture medium (Co et al., 2019; Li et al., 2020; Nash et al., 2021; Kakni et al., 2022a). To generate apical-out intestinal organoids from PSCs, we utilized a previously described stepwise differentiation method (Kakni et al., 2022a) (Figure 1A). In contrast to previous methods, our PSC-derived apical-out organoids were solely cultured in suspension and were not embedded in Matrigel in any step during the differentiation period. Briefly, H9 embryonic stem cells were seeded into microwell arrays (1,000 cells/microwell) in order to create homogenous embryoid bodies, which were then differentiated towards definitive endoderm and hindgut without disrupting their 3D conformation. Subsequently, hindgut spheroids were removed from the microwell arrays and transferred to a suspension culture system in order to further mature to intestinal organoids with reversed polarity (Supplementary Figure S1). This system has great potential to generate large numbers of organoids, since 289 homogenous hindgut spheroids, which will further mature into intestinal organoids, can be harvested from each microwell array. This number corresponds to almost 7,000 organoids from a single 24-well plate. Thus, this system facilitates scaled-up production of apical-out organoids, which can be used in high-throughput applications, such as drug screenings.

One of the key functions of the intestinal epithelium is the formation and maintenance of a selective barrier, which allows the passage of essential nutrients but prevents the passage of harmful external factors, such as harmful microbes and toxins. This balance is also essential for the proper function of the intestinal epithelial cells. Special protein complexes are responsible to interconnect the individual cell membranes in order to maintain this barrier function and seal the intercellular space, while adhering the neighbor cells keeping them as an epithelial sheet. These include the desmosomes, adherent junctions and tight junctions (Groschwitz and Hogan, 2009; Chelakkot et al., 2018). These complexes have been previously identified in intestinal organoids (Pearce et al., 2018). Here, we aimed to explore whether such structures are present in intestinal organoids with reversed polarity. After establishing the inside-out intestinal organoids, we looked into their ultrastructural organization in order to identify the junctional complexes that are responsible for the cell adhesion and the barrier formation and maintenance (Figure 1B). In a panoramic view of the middle and apical region of an enterocyte, we identified microvilli in the outer surface of the organoid, showing a well-developed glycocalix and actin bundles deep in the terminal web area (Figure 1B). Mitochondria, smooth and rough endoplasmic reticulum (ER) and Golgi complex appear very similar in morphology and localization to those present in the intestinal epithelium (Sesorova et al., 2020). Additionally, junctional complexes including tight junctions, zonula adherens and desmosomes are present in the apical side of the cells. Basement membrane, mitochondria and collagen fibers were identified in the basal region. Taken together, the observations indicate that our apical-out intestinal organoids recapitulate fully functional enterocytes.

To further assess the presence of junctional complexes, we performed gene expression analysis for zonula occludens 1 (*ZO-1*) and occludin (*OCLN*), which regulate the “leak pathway” that mediates the flux of large molecules (up to 6 nm) and claudin-1, 3 and 5 (*CLDN1, CLDN3, CLDN5*), which are responsible for the “pore pathway” that mediates the movement of small ions and solutes (up to 0.8 nm) (Pearce et al., 2018; Monaco et al., 2021). In all cases, there was a significant increase in the expression levels compared to undifferentiated stem cells, thus further confirming that apical-out intestinal organoids harbor functional junctional complexes (Figure 1C).

**FIGURE 2:**
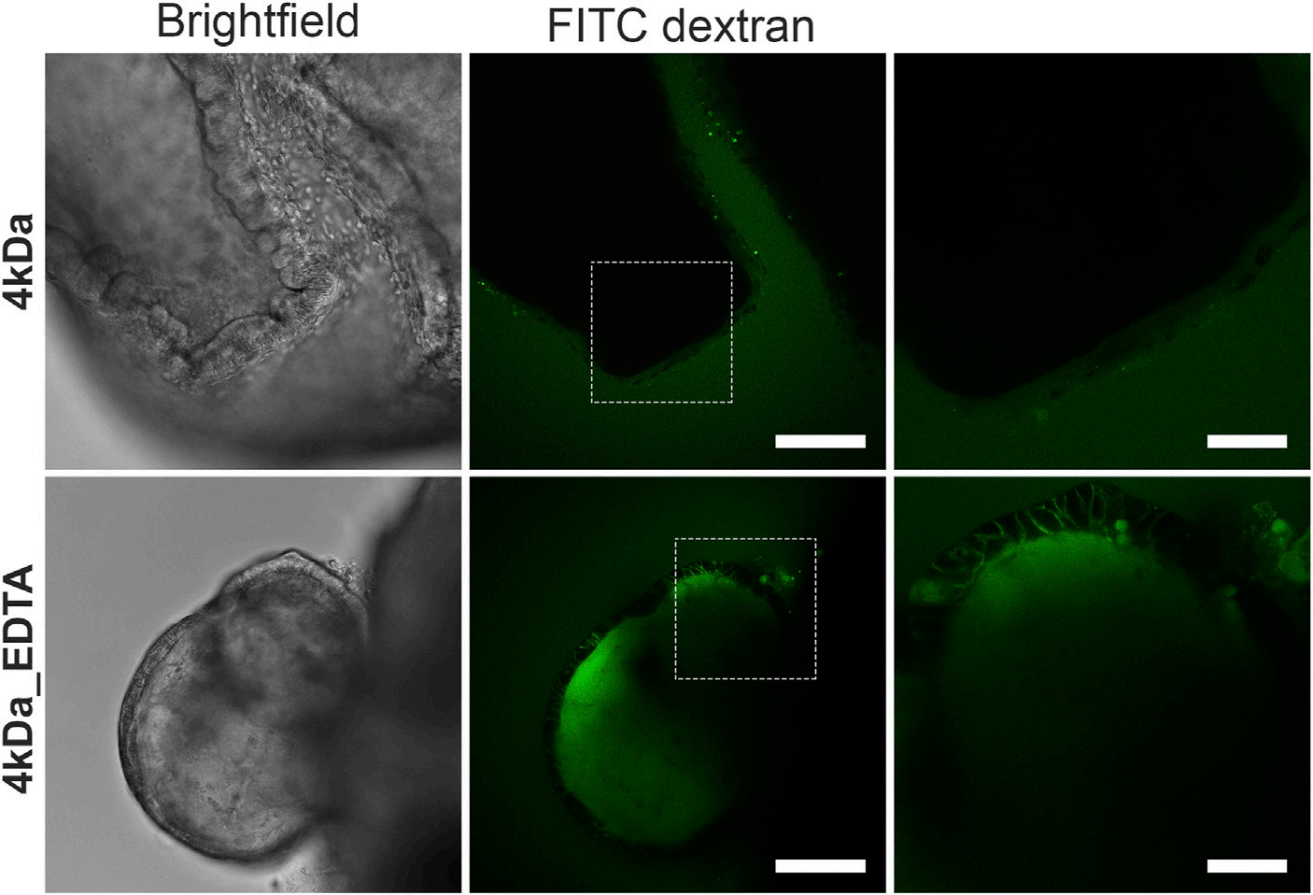
Suspension intestinal organoids show epithelial barrier integrity by a FITC-dextran diffusion assay. Suspension organoids exclude the 4 kDa FITC-dextran, showing tight epithelial barrier formation (top row). When organoids are treated with 2 mM EDTA (bottom row), the barrier is disrupted and FITC-dextran diffuses into the intracellular space and the organoid lumen. White boxes in the middle images delineate the areas shown in the zoomed images to the respective right. Scale bars: 100 μm (left and middle) and 50 μm (right).

To determine the integrity of the organoid barrier, we performed a FITC-dextran diffusion assay, which has been used extensively to assess gut barrier integrity and permeability both in *in vivo* (Baxter et al., 2017; Woting and Blaut, 2018) and *in vitro* (Kowapradit et al., 2010; Xu et al., 2019) systems. In our case, FITC-dextran of 4 kDa (FITC-D4) was added to the organoid culture medium for 30 min and its diffusion into the lumen of the organoids was observed using confocal microscopy (Figure 2). The apical-out intestinal organoids excluded entirely the FITC-D4, thus demonstrating strong epithelial barrier integrity. As a positive control, organoids were treated with 2 mM EDTA for 15 min, which is a chelating agent known to disrupt the tight junctions by depleting calcium in the medium and thus increasing permeation of compounds through the paracellular route (Chung et al., 1998; Zheng et al., 2006). In EDTA-treated organoids, the FITC-D4 diffused into the intercellular spaces and lumen of the organoids, thus showing a compromised epithelial barrier. These findings strongly support that apical-out intestinal organoids form an effective barrier that recapitulates multiple features of the *in vivo* intestinal epithelial barrier.

### 3.2 Nutrient transport and metabolism

**FIGURE 3:**
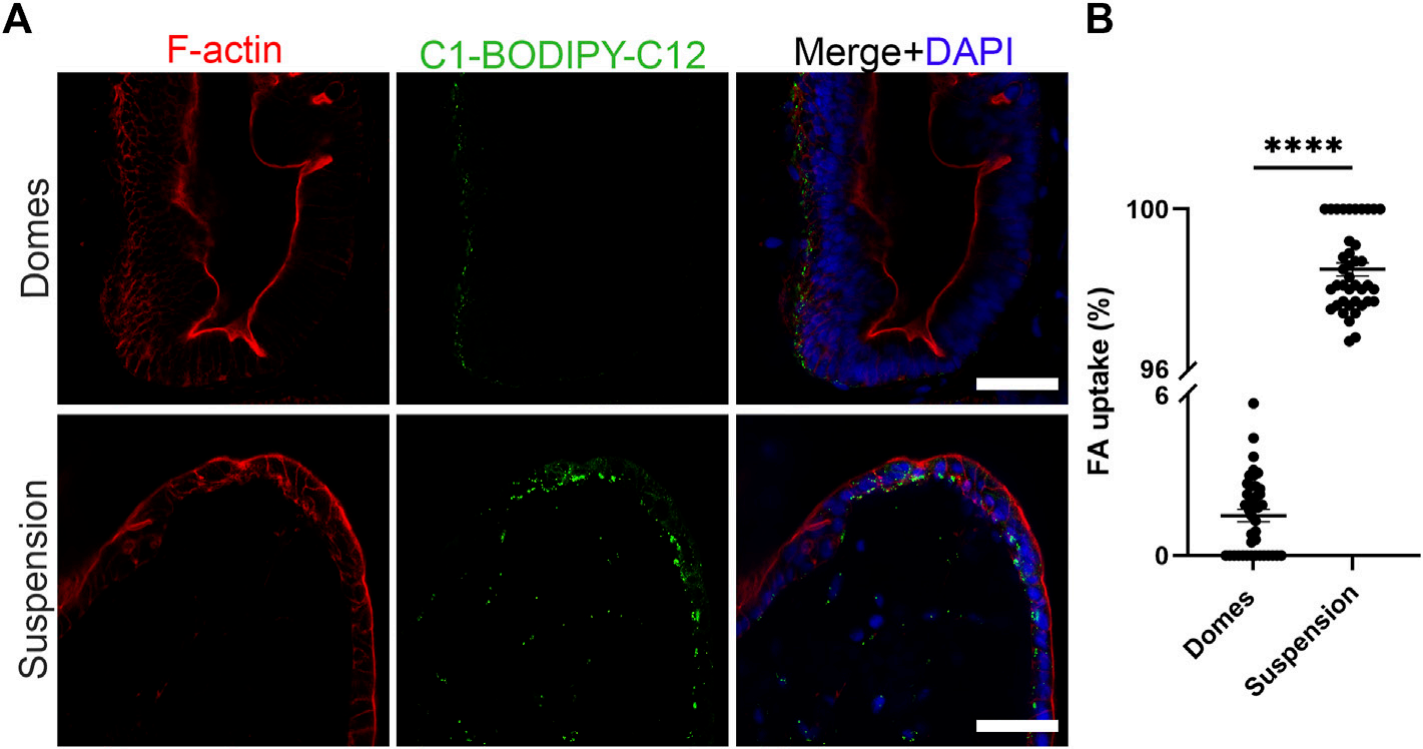
Suspension intestinal organoids readily absorb fatty acids. **(A)** The fluorescent fatty acid (FA) analog C1-BODIPY-C12 (green) is only taken up when the apical surface of the organoids is facing outwards. Phalloidin (red) marks the apical side of the epithelium and DAPI (blue) the nuclei. Scale bars: 50 μm, and apply to images in the respective row. **(B)** Quantification of the FA uptake in domes and suspension intestinal organoids. The percentage of FA uptake corresponds to the amount of measured fluorescence in the intercellular space, over the total fluorescence. Error bars indicate mean ± S.E.M. (*n* = 4).

The intestinal epithelium is a highly polarized cell layer that plays a pivotal role in nutrient absorption and metabolism. The uptake of fatty acids in the intestine occurs through fatty acid transporters that are located at the apical side of the enterocytes (Wang et al., 2013). Once inside the cells, fatty acids are transported to the endoplasmic reticulum where they are used for triglyceride synthesis. Triglycerides are packaged with lipoproteins, cholesterol and other lipids into chylomicrons. These particles are secreted from the enterocytes into the lymph across the basolateral membranes of the cells (McLeod and Yao, 2016). To assess the polarity-specific fatty acid absorption, we used the fluorescent fatty acid analog C1-BODIPY-C12 (Figure 3) (Co et al., 2019). We incubated both apical-in and apical-out organoids with 5 μM BODIPY dye for 30 min and then we fixed and stained the organoids with phalloidin (indicating F-actin) and DAPI (indicating cell nuclei). Confocal microscopy showed strong BODIPY signal in the apical-out organoids, demonstrating the successful absorption of the fatty acid analog from the surrounding medium. In comparison, in apical-in organoids, the BODIPY signal was significantly weaker and located in the outer surface of the organoids, indicating that fatty acids are not taken up from the medium and lipid droplets did not form. These data suggest that in apical-out organoids, the apical fatty acid transporters are directly accessible in the outer surface of the organoids.

**FIGURE 4:**
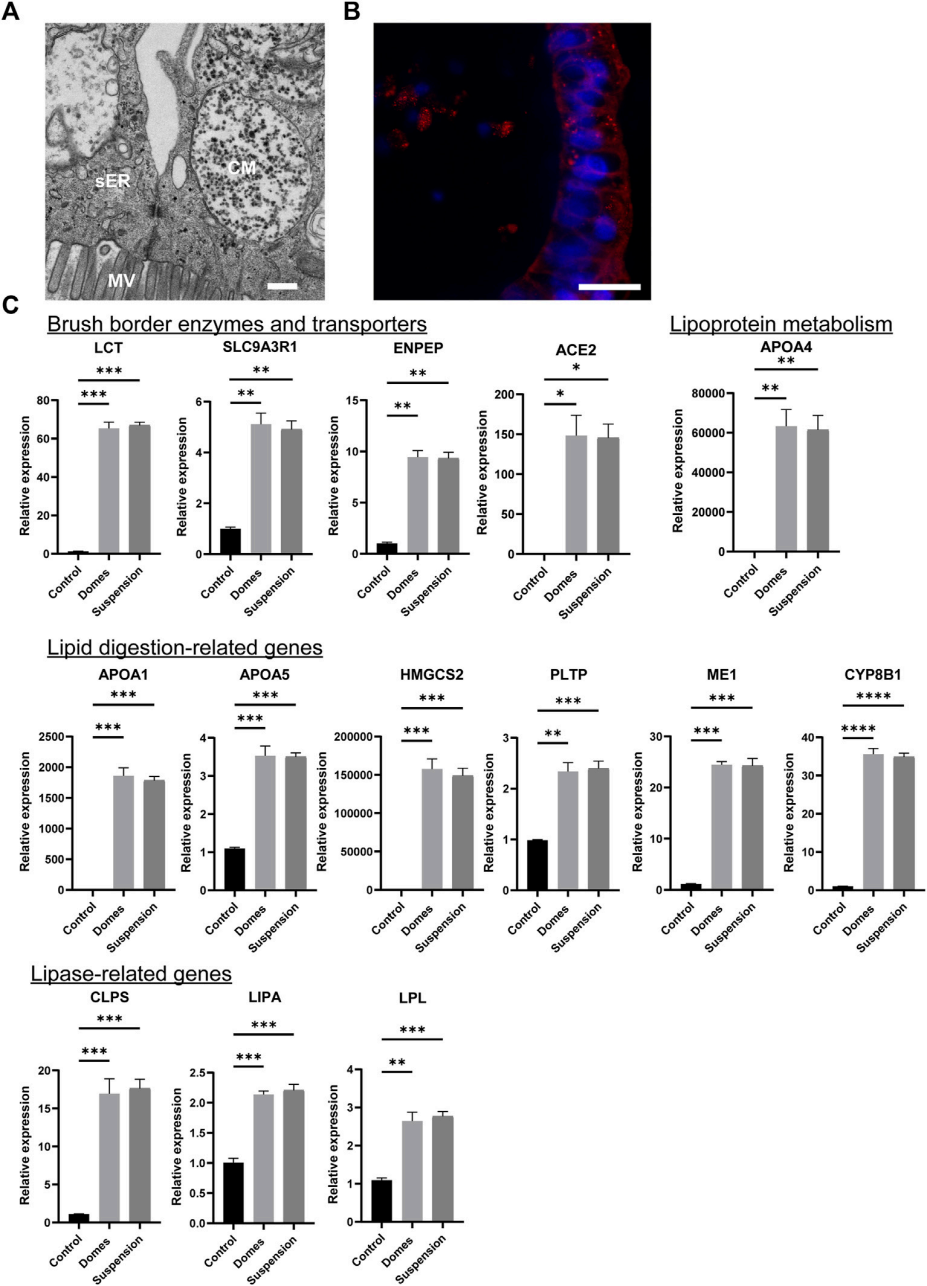
Suspension intestinal organoids recapitulate *in vivo* metabolic activity. **(A)** TEM demonstrates the presence of chylomicrons (CM) and vesicles of smooth endoplasmic reticulum (sER) in apical-out organoids. MV: microvilli. Scale bar: 500 nm. **(B)** Nile red staining indicating the lipid droplets in the cytosol of apical-out intestinal organoids. Scale bar: 20 μm. **(C)** qRT-PCR analysis demonstrates the expression levels of the brush borders enzymes and transporters (*LCT, SLC9A3R1, ENPEP, ACE2*); the lipoprotein metabolism-related gene (*APOA4*); the lipid digestion-related genes (*APOA1, APOA5, HMGCS2, PLTP, ME1, CYP8B1*) and the lipase-related genes (*CLPS, LIPA, LPL*) in both apical-in (domes) and apical-out (suspension) intestinal organoids. Untreated H9 embryonic stem cells were used as controls. Error bars indicate mean ± S.E.M (*n* = 3).

Next, we assessed whether the apical-out organoids can perform intestinal metabolic functions. First, we determined whether the apical-out organoids harbor chylomicrons and vesicles of smooth endoplasmic reticulum, which are involved in processing absorbed fatty acids and monoglycerides *in vivo*. TEM analysis indeed demonstrated the presence of these structures (Figure 4A). Nile red staining indicated the formation of lipid droplets in the cytosol of the apical-out organoids (Figure 4B). Cytosolic lipid droplets are organelles found in most tissues and they play a crucial role in energy storage, inter-organelle communication and cellular metabolic processes (Auclair et al., 2018). Then, we performed gene expression analysis for 14 different lipid metabolism markers (Figure 4C). First, we investigated the expression of brush border enzymes and transporters, including Lactase (*LCT*), Sodium-hydrogen antiporter 3 regulator 1 (*SLC9A3R1*), Glutamyl aminopeptidase (*ENPEP*) and Angiotensin-converting enzyme 2 (*ACE2*). Additionally, we assessed the expression of the lipoprotein metabolism-related gene *APOA4* and the expression of lipid digestion-related genes, such as the apolipoproteins A1 and 5 (*APOA1*, *APOA5),* the 3-hydroxy-3-methylglutarylCoA synthetase 2 (*HMGCS2*), the Phospholipid transfer protein (*PLTP*), the Cytosolic malic enzyme 1 (*ME1*) and the sterol 12-alpha-hydroxylase (*CYP8B1*). Finally, we evaluated the expression of lipase-related genes, including the Colipase (*CLPS*), lipase A (*LIPA*) and Lipoprotein lipase (*LPL*). Further information regarding the functions of these genes can be found in the Supplementary Table S4. The expression of all these genes was significantly increased in both apical-in and apical-out mature intestinal organoids when compared to undifferentiated stem cells. However, no significant difference was observed in gene expression levels between the two organoid models, indicating that our new apical-out intestinal organoids recapitulate intestinal metabolic functions similar to the already established apical-in intestinal organoids.

### 3.3 Apical-out organoids express drug-metabolizing enzymes and drug transporters

Apart from its key role in nutrient absorption, the intestine is involved in drug metabolism of orally administered drugs. Drug-metabolizing enzymes of the intestine contribute to first-pass drug metabolism and are responsible for the low oral bioavailability of numerous compounds (Fritz et al., 2019). So far, cell lines (e.g., Caco-2) have been utilized on either culture inserts or on-chip systems for drug absorption studies (Youhanna and Lauschke, 2021). Organoids have not been widely used in such studies, mainly due to the limited accessibility of the lumen, which makes it more complicated to mimic a physiologically relevant drug transport (Youhanna and Lauschke, 2021). However, apical-out intestinal organoids have the potential to overcome this technical difficulty and can become a valuable new tool for *in vitro* drug testing. Previous studies have shown that intestinal organoids express certain drug metabolizing enzymes and transporters (Negoro et al., 2018; Kondo et al., 2020; Sasaki et al., 2021). Here, we aimed to explore whether the apical-out intestinal organoids express these enzymes and transporters at similar levels as apical-in organoids.

**FIGURE 5:**
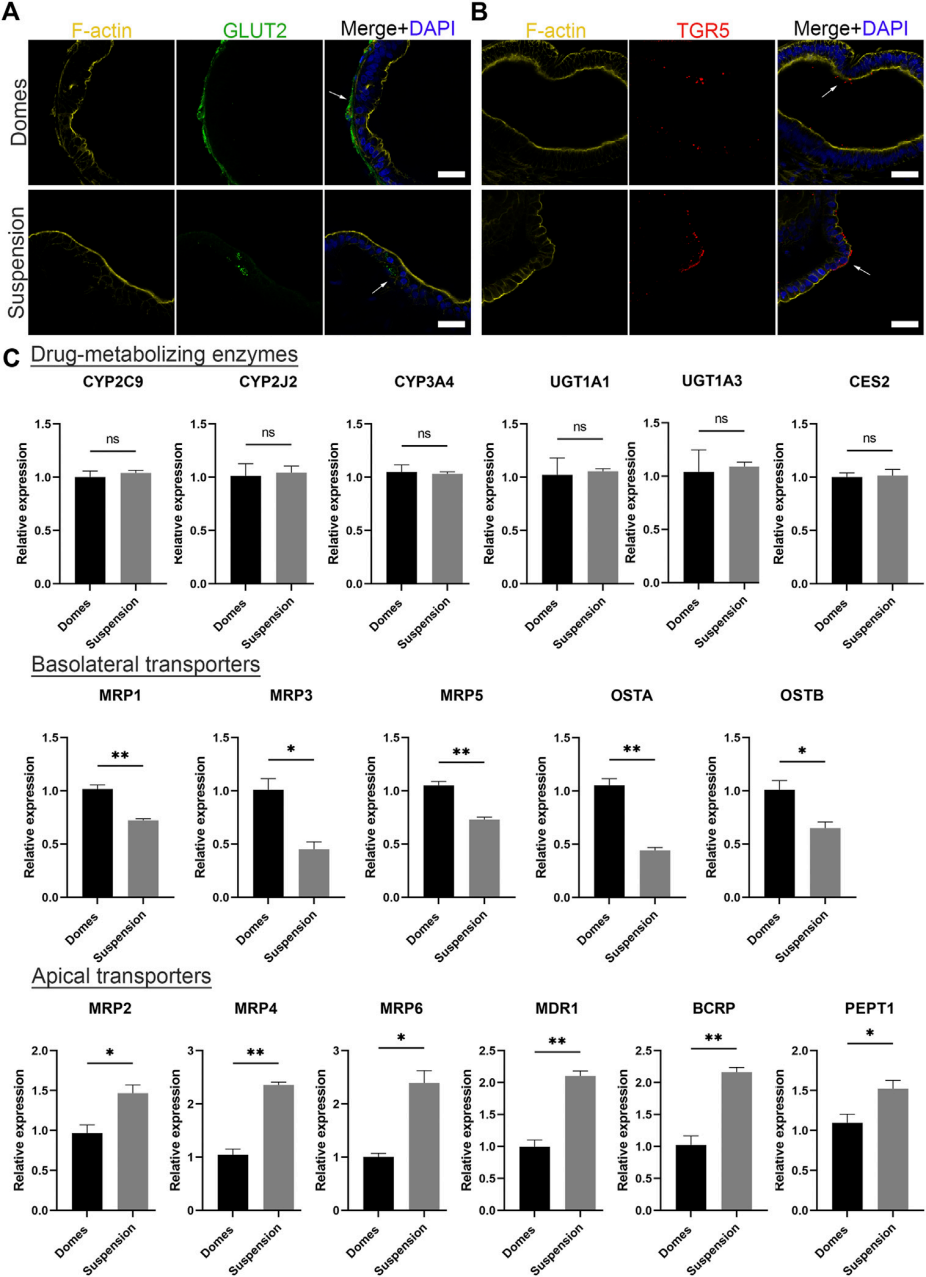
Suspension intestinal organoids express drug-metabolizing enzymes and transporters. **(A, B)** Immunofluorescence stainings of the basal transporter GLUT2 (green) and the apical bile acid receptor TGR5 (red) in domes (top) and suspension (bottom) organoids. Phalloidin (yellow) indicates F-actin and DAPI (blue) indicates cell nuclei. Scale bars: 50 μm, and apply to the same group in each row. **(C)** Gene expression levels encoding drug-metabolizing enzymes (*CYP2C9, CYP2J2, CYP3A4, UGT1A1, UGT1A3 and CES2*), basolateral transporters (*MRP1, MRP3, MRP5, OSTA and OSTB*) and apical transporters (*MRP2, MRP4, MRP6, MDR1, BCRP and PEPT1*) were measured by real time RT-PCR. Error bars indicate mean ± S.E.M (*n* = 3).

Initially, to visualize the position of apical and basal transporters, we performed immunofluorescence stainings against the basal glucose transporter 2 (GLUT2) and the apical bile acid receptor: Takeda G-protein-coupled receptor 5 (TGR5) (Figure 5A). The expression of GLUT2 was identified in the outer surface of apical-in organoids and in the inner surface of apical-out organoids. Conversely, TGR5 was found in the inner part of apical-in and in the outer part of apical-out organoids. These data are consistent with previous studies with apical-in intestinal organoids (Zietek et al., 2015) and indicate that the transporters still show correct basal and apical localizations in apical-out organoids. Following that, we evaluated the gene expression levels of drug-metabolizing enzymes and transporters in apical-in and apical-out organoids using quantitative RT-PCR (Figure 5B). Specifically, we assessed the expression of the cytochrome P450 family 2 subfamily C member 9 (*CYP2C9*), cytochrome P450 family 2 subfamily J member 2 (*CYP2J2*), *CYP3A4*, UDP glucuronosyltransferase family one member A1 (*UGT1A1*), UDP glucuronosyltransferase family one member A3 (*UGT1A3*), and carboxylesterase 2 (*CES2*). In both organoid models, these enzymes are expressed at similar levels without statistically significant differences. Next, we assessed the expression of several basolateral transporters. These include the multidrug resistance-associated protein 1, 3 and 5 (*MRP1, MRP3, MRP5*) and the organic solute transporter alpha and beta (*OSTA, OSTB*). Interestingly, in apical-in organoids (domes), the expression of these transporters was significantly higher compared to apical-out organoids (suspension). Then, we investigated the expression of the apical transporters: *MRP2, MRP4, MRP6,* multidrug resistance protein 1 (*MDR1*), breast cancer resistance protein (*BCRP*) and peptide transporter 1 (*PEPT1*). Unlike the basolateral transporters, the expression levels of the apical transporters were significantly higher in apical-out organoids.

**FIGURE 6:**
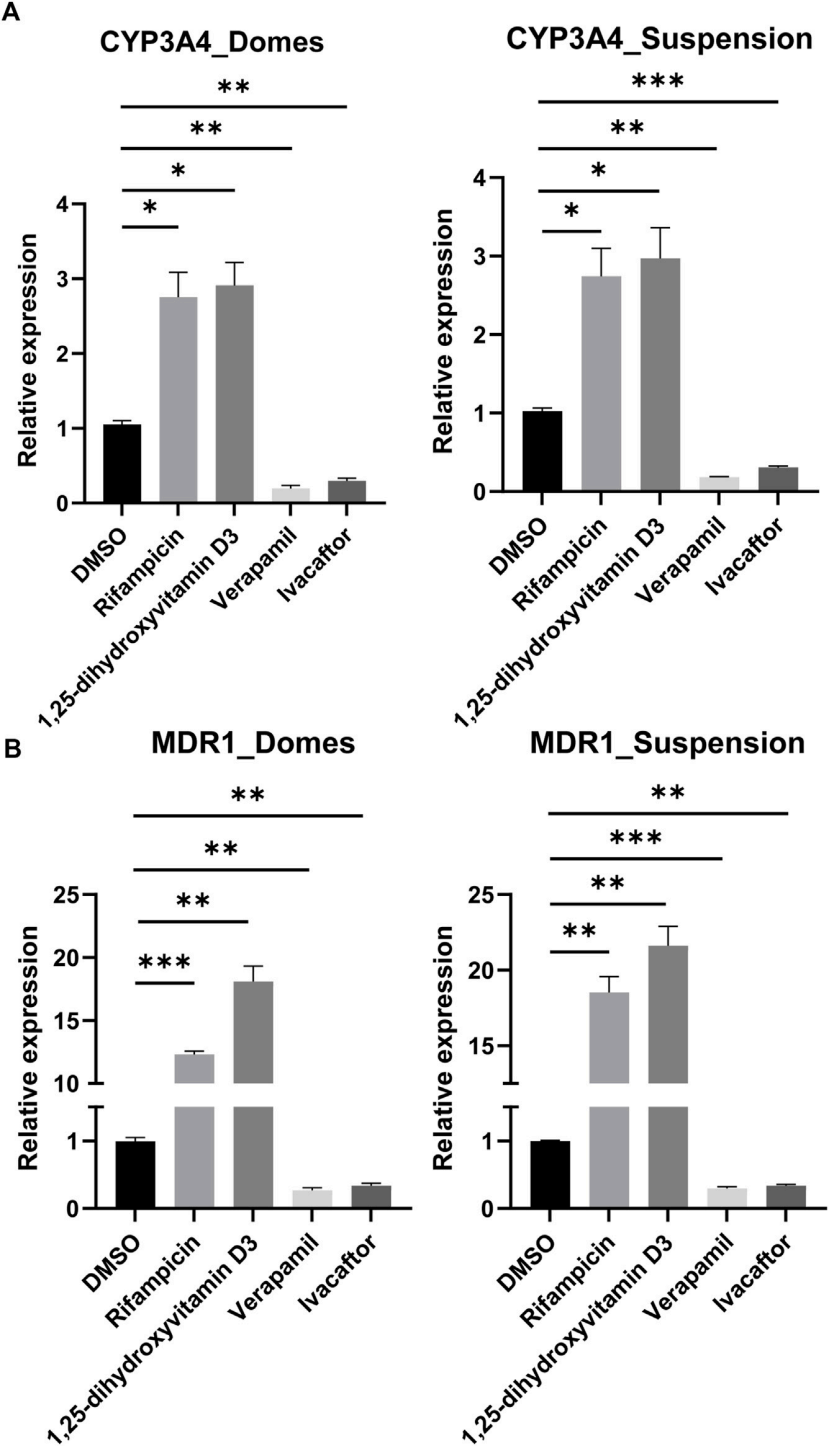
Induction and inhibition of *CYP3A4* and *MDR1*. **(A, B)** Assessment of *CYP3A4* **(A)** and *MDR1* **(B)** levels in organoids grown embedded in Matrigel domes and suspension, following drug treatment with the inducers rifampicin and 1,25-dihydroxyvitamin D3 and the inhibitors verapamil and ivacaftor for 48 h. In both organoid models, there are significant differences in the expression levels following treatments compared to DMSO-treated controls. Notably, the upregulation of *MDR1* expression following treatment with rifampicin and 1,25-dihydroxyvitamin D3 was more robust in the apical-out organoids than in the apical-in organoids. Error bars indicate mean ± S.E.M (*n* = 3).

After having confirmed that *CYP3A4* and *MDR1* are expressed in both apical-in and apical-out organoids, we aimed to assess their activity following drug treatments. *CYP3A4* is one of the most dominant and abundant drug-metabolizing enzymes (Negoro et al., 2018) and *MDR1* (also known as P-glycoprotein or *ABCB1*) is actively involved in the transport of drugs and the modulation of the intracellular concentration of toxic compounds and drug components (Hoffmann and Kroemer, 2004). Induction of these enzymes is one of the major concerns for pharmacokinetic studies since they affect the oral bioavailability of drugs. We exposed these organoids to rifampicin and 1,25-dihidroxyvitamin D3, which are both known inducers of *CYP3A4* and *MDR1* (Figures 6A, B)*.* In both organoid models, there was a significant increase in the expression of *CYP3A4* and *MDR1* compared to DMSO-treated controls*.* In addition, we treated the organoids with the inhibitors verapamil and ivacaftor (weak inhibitor) (Figure 6A, B)*.* In this case, we found a significant decrease in the expression of both *CYP3A4* and *MDR1* compared to DMSO-treated controls*.* Collectively, these results indicate that the drug-metabolizing enzymes and transporters are not just present in the apical-out intestinal organoids, but they are also functional.

## 4 Discussion

In this study, we demonstrated that human PSC-derived intestinal organoids with apical-out orientation can successfully perform specialized intestinal functions, such as barrier formation, polarized nutrient uptake and drug absorption and metabolism. Exceptional advantages of this state-of-the-art model include the scalability of the system; approximately 7,000 organoids can be derived from a single 24-well plate and the ability to generate organoids from various cell sources including both embryonic and induced PSCs (iPSCs) (Kakni et al., 2022a). Considering that iPSCs can be generated from patients with various diseases, apical-out organoids derived from these cells could further contribute to the field of disease modeling and personalized medicine, since various and combinatorial drug-treatments could be tested in simplified 3D assays in a high-throughput manner. So far, the front-runners for such studies were cancer-derived cell lines such as Caco-2. These cells are predominating the *in vitro* studies of differentiation, permeability and absorption since they are easy to culture, cheap to use and spontaneously differentiate into a polarized epithelium with typical finger-like villi, upon reaching confluence (Fois et al., 2019). However, these cells are cultured as monolayers and usually recapitulate only the enterocytes. Thus, the 3D architecture and the multicellular composition of the *in vivo* intestine are not properly represented. These key *in vivo* features are fundamental for the proper function of the intestine; hence, organoids mimicking those can be superior models for the performance of functional assays to Caco-2 cells. To increase the complexity of such 2D systems and better recapitulate the *in vivo* intestine, various microfluidic devices have been developed which allow for fluid flow, peristalsis-like motions and/or formation of villi structures (Bein et al., 2018; Xiang et al., 2020). *In vitro* models incorporating such features have greatly benefited studies related to intestinal functions, such as the absorption, distribution, metabolism and excretion of nutrient and drugs. However, the majority of these models have been developed for Caco-2 cells. Only recently, microfluidic devices were developed for intestinal organoid-derived cell monolayers (Kasendra et al., 2018; Jalili-Firoozinezhad et al., 2019; Kasendra et al., 2020). These models demonstrated successful formation of crypt-villus structures, multicellular composition and barrier integrity, and were proven valuable for drug discovery and host-microbiome interaction studies. Even though these platforms are a great advancement comparing to the Caco-2 models, organoids still lose their 3D architecture and a high number of cells is required to create monolayers, which translates to increased costs, since more organoids are needed. Additionally, the use of microfluidic devices complicates high-throughput applications and more technological advancements are required to increase the compatibility with such assays (De Stefano et al., 2022). Our apical-out intestinal organoid model overcomes some of these limitations and shows great potential for nutrient and drug uptake and metabolism studies.

Looking into the gut epithelial barrier function in apical-out organoids, we identified all the junctional complexes that are responsible for the formation of a selective barrier. Specifically, we detected desmosomes, tight junctions and zonula adherens in the apical surface of the organoids, similar to the *in vivo* intestine (Chelakkot et al., 2018). The FITC-dextran diffusion assay demonstrated the tightness of our reversed-orientation epithelium. This is in accordance with previous publications on intestinal organoids with such an apical-out architecture (Co et al., 2019; Nash et al., 2021). Collectively, these results showed that apical-out organoids not only form a tightly sealed barrier but also have the “means” to allow selective transport of molecules. Increased intestinal permeability has been associated with numerous diseases including inflammatory bowel disease, irritable bowel syndrome, diabetes and Alzheimer’s disease (Vanuytsel et al., 2021). This reversed polarity intestinal organoid model can be a valuable tool to evaluate intestinal permeability *in vitro*, thus assisting the detection of relevant diseases and later their treatment, since it can be used as (personalized) drug-screening platform. The exposure of the directly accessible apical surface to test probes (i.e., dextrans, polyethylene glycol of different molecular weights) can facilitate the process compared to the formation of 2D monolayers from organoid cells (Altay et al., 2019) or microinjections in the lumen of apical-in organoids (Hill et al., 2017; den Daas et al., 2022). However, it is worth mentioning that performing measurements like transepithelial electrical resistance (TEER) and apparent permeability (Papp) cannot be performed in such apical-out organoids. Additionally, the average size of most drugs is 0.1 kDa–1 kDa and the FITC-dextrans usually used to assess barrier integrity are much bigger (4 kDa–40 kDa). A potential alternative molecule to further study epithelial integrity and confirm our results is Lucifer yellow. In future studies, it would be useful to develop new assays with fluorescent compounds that better mimic average size of the drugs.

Almost all nutrients are absorbed and transported *via* the highly polarized intestinal epithelium *in vivo*. Fatty acids are absorbed from the lumen through the apical surface of the intestinal absorptive cells (Wang et al., 2013). We demonstrated here that in organoids where the apical side is facing outwards, the fluorescent fatty acid analog BODIPY was absorbed through the apical surface whereas this was not observed in apical-in organoids (Figure 3). Similar results were previously shown for reversed polarity organoids derived from adult stem cells (Co et al., 2019). We took this finding a step further and demonstrated that these organoids recapitulate key metabolic functions. With a closer look into the ultrastructural organization of the apical-out organoids, we identified chylomicrons and vesicles of smooth endoplasmic reticulum, which indicate active processing of the dietary fats that are taken up by enterocytes. We also identified cytosolic lipid droplets that store the excess dietary triacylglycerols. Moreover, gene expression analysis showed also the presence of brush border enzymes and transporters, lipoprotein metabolism genes, lipid digestion–related genes and lipase-related genes, which map to the peroxisome proliferator-activated receptor (PPAR) signaling pathway, a key regulator of intestinal metabolism (de Vogel-van den Bosch et al., 2008; Duszka et al., 2016; 2017). Taken together, these data confirm that apical-out intestinal organoids can successfully recapitulate nutrient uptake and metabolic functions, which are major functions of the intestine. These intestinal functions profoundly affect the proper function of the whole body, since the availability and quality of nutrients are translated to substrates that are distributed to every organ in order to supply energy (Ko et al., 2020). Nutrient transport and metabolism have not been widely studied in organoids since the architecture with an enclosed central lumen does not provide easy access to the apical surface, which is the nutrient absorption site. Therefore, our reversed polarity organoid model can further enhance the study of nutrient absorption, transport and metabolism because, by simply adding nutritional compounds to the culture medium, a physiologically relevant nutrient uptake *via* the apical surface and subsequent metabolism can be mimicked in a 3D organoid context.

The vast majority of drugs are administered orally, thus, it is important to predict their bioavailability. Absorption, distribution, metabolism and excretion are the processes that need to be studied in order to determine the bioavailability (Yu et al., 2014; Fedi et al., 2021). Here, we demonstrated that intestinal organoids with reversed polarity express drug metabolizing enzymes similar to established apical-in intestinal organoids (Negoro et al., 2018; Kondo et al., 2020). However, we found a higher expression of apical transporters in apical-out organoids and higher expression of basolateral transporters in apical-in organoids (Figure 5C). The expression of these transporters can be affected by endogenous and exogenous compounds, such as nutrients and hormones (Takano et al., 2006; Arana et al., 2016). Therefore, we hypothesize that the differences in the expression levels of transporters between apical-in and apical-out organoids are mainly caused by the different concentrations of substances present in the enclosed organoid lumen or in the surrounding cell culture medium. Further studies would be required to shed light on the mechanisms underlying these gene expression differences, but this is beyond the scope of this paper. It is noteworthy that organoids derived from PSCs are generally less functionally mature than ASC-derived organoids and *in vivo* tissues and usually resemble closer the fetal tissues (McCauley and Wells, 2017; Taelman et al., 2022). Thus, we expect lower expression levels of these transporters and drug metabolizing enzymes in our apical-out PSC-derived organoids, comparing to the *in vivo* intestine. CYP3A4 is a dominant drug metabolizing enzyme and MDR1 plays an important role in intestinal absorption and excretion of drugs (Negoro et al., 2018). Treatment with rifampicin (Kolars et al., 1992) and 1, 25-dihidroxyvitamin D3 (Theodoropoulos et al., 2003) induced the expression of CYP3A4 and MDR1 in both apical-in and apical-out organoids (Negoro et al., 2018; Onozato et al., 2018; Kondo et al., 2020). In contrast, treatment with verapamil (Zhao et al., 2017) and ivacaftor (Robertson et al., 2015) reduced their expression as suggested by their inhibitory role. To our knowledge, this is the first report showing that apical-out intestinal organoids contain functional drug metabolizing enzymes and transporters, which respond to drug treatments that were previously studied in apical-in organoids or 2D monolayer models. This organoid model is of great importance for future drug studies, since organoids are more physiologically relevant models than 2D cultures and the human cell origin can overcome the species-specific differences that arise with animal-based testing, ultimately leading to faster and more successful drug development.

To conclude, we have used a scalable apical-out intestinal organoid model, derived from hPSCs, and demonstrated their ability to perform specialized intestinal functions. These organoids are cultured solely in suspension and form a tight barrier, perform polarized nutrient absorption and lipid metabolism and express active drug metabolizing enzymes and transporters. The direct access to the apical surface of the organoids facilitate nutrient and drug absorption studies, since the tested substances can be simply added to the medium, bypassing the need for microinjections and gel diffusion. Therefore, this platform can be a timesaving and cost-efficient method for high-throughput and animal-free nutrition and drug discovery studies in the future.

## Data Availability

The original contributions presented in the study are included in the article/Supplementary Material, further inquiries can be directed to the corresponding author.
