# Supplementary material for: PSC-derived intestinal organoids with apical-out orientation as a tool to study nutrient uptake, drug absorption and metabolism
Source: Front Mol Biosci. 2023 Jan 18;10:1102209. doi: 10.3389/fmolb.2023.1102209 (PMC9889654; doi:10.3389/fmolb.2023.1102209)
Supplement: Supplementary file 1 [file DataSheet1.PDF]

Supplementary Material

1 Supplementary Tables

1.1 Supplementary Table 1. List of catalog numbers for the materials of this study

Material	Company	Catalog number
1,25-dihydroxyvitamin D3	Sigma-Aldrich	D1530
2-propanol	VWR	20922.320
Activin A	Cell Guidance Systems	GFH6-100
B27	Thermofisher	12587010
C1-BODIPY-C12	Thermofisher	D3823
Cacodylate	Acros Organics	318150050
CHIR99021	Stemgent	04-0004-02
DMEM-F12	Thermofisher	11320033
DMSO	VWR	A3672.0250
EDTA	VWR	324504-500
EGF	R&D systems	236-EG-200
Ethanol	VWR	8.18760.2500
Fatty-acid-free BSA	Sigma-Aldrich	A8806
Ferricyanide	Merck	702587
FGF4	R&D Systems	235-F4-025
FITC-labeled dextran	Sigma-Aldrich	46944
Formaldehyde	VWR	20910.294
Gentle Cell Dissociation Reagent	STEMCELL Technologies	07174
Glutaraldehyde	Merck	G7651
H9 cells	WiCell	WA09
HBSS (no calcium and no magnesium)	Thermofisher	14175095
Hepes	Thermofisher	15-630-106
HyClone defined fetal bovine serum	Thermofisher	11591821
iScript cDNA Synthesis Kit	Bio-Rad	1708891
Ivacaftor	Selleck Chemicals	S1144
L-glutamine	Thermofisher	25-030-081
Matrigel® Basement Membrane Matrix, LDEV-free	Corning	354234
Matrigel® hESC-Qualified Matrix, LDEV-free	Corning	354277
mTeSR™1	STEMCELL Technologies	85850_C
N2	Thermofisher	17502048
Nile red	Sigma-Aldrich	19123
Noggin	R&D systems	6057-NG-100
O-rings	Eriks	10023241
Osmium tetroxide	Agar Scientific	AGR1015
PBS	Sigma-Aldrich	D8537
Penicillin/Streptomycin	Thermofisher	15-140-122
Pluronic F-108	Sigma-Aldrich	542342
Primers	Sigma-Aldrich	
Rifampicin	Sigma-Aldrich	R3501
RNeasy Mini Kit	Qiagen	74104

RPMI 1640	Thermofisher	11-875-093
R-Spondin	R&D systems	4645-RS
Sucrose	Merck	S1888
Triton X-100	Sigma-Aldrich	10789704001
TrypLE™ Express Enzyme	Thermofisher	12604013
Verapamil	Sigma-Aldrich	V4629
Y-27632	Tocris	1254/10

1.2 Supplementary Table 2. Primer sequences

Gene	5'–Forward– 3'	5'–Reverse– 3'
ACE2	CAAGAGCAAACGGTTGAACAC	CCAGAGCCTCTCATTGTAGTCT
APOA1	CCCTGGGATCGAGTGAAGGA	CTGGGACACATAGTCTCTGCC
APOA4	CTCAAGGGACGCCTTACGC	GTCCTGAGCATAGGGAGCCA
APOA5	GCCAGCGACTTCAGGCTTT	AGCTTGCTCAGAACCTTGCC
BCRP	ACGAACGGATTAACAGGGTCA	CTCCAGACACACCACGGAT
CES2	CATGGCTTCCTTGTATGATGGT	CTCCAAAGTGGGCGATATTCTG
CLDN1	CCCAGTCAATGCCAGGTACG	GGGCCTTGGTGTTGGGTAAG
CLDN3	AACACCATTATCCGGGACTTCT	GCGGAGTAGACGACCTTGG
CLDN5	GCAGCCCCTGTGAAGATTGA	GTCTCTGGCAAAAAGCGGTG
CLPS	CTCTGCATGAATAGTGCCCAG	AGGGACACTTGTAGTAAATCCCA
CYP2C9	CAGAGACGACAAGCACAACCCT	ATGTGGCTCCTGTCTTGCATGC
CYP2J2	TGGCTTGCCCTTAATCAAAGAA	GGCCACTTGACATAATCAATCCA
CYP3A4	AAGTCGCCTCGAAGATACACA	AAGGAGAGAACACTGCTCGTG
CYP8B1	ATTTGGATACCGTTCAGTGCAA	CAGAAGCGAAAGAGGCTGTC
ENPEP	CTTGACCAGATCGTGTGACTC	GGCAGTCGAAAGTTTTTCCAC
HMGCS2	CAGTCCAAGAGGACATCAACTC	CAGTGCCTACTTCCAGCCTG
LCT	ATCCAGACGAGAAAACAGTGC	GTCAGCAAAGGCTTCGGTTC
LIPA	CCCACGTTTGCACTCATGTC	CCCAGTCAAAGGCTTGAAACTT
LPL	TCATTCCCGGAGTAGCAGAGT	GGCCACAAGTTTTGGCACC
MDR1	GGGATGGTCAGTGTTGATGGA	GCTATCGTGGTGGCAAACAATA
ME1	GGGAGACCTTGGCTGTAATGG	TTCGGTTCCCACATCCAGAAT
MRP1	TTACTCATTCAGCTCGTCTTGTC	CAGGGATTAGGGTCGTGGAT
MRP2	TCTCTCGATACTCTGTGGCAC	CTGGAATCCGTAGGAGATGAAGA
MRP3	CACCAACTCAGTCAAACGTGC	GCAAGACCATGAAAGCGACTC
MRP4	TGTGGCTTTGAACACAGCGTA	CCAGCACACTGAACGTGATAA
MRP5	GAACTCGACCGTTGGAATGC	TCATCCAGGATTCTGAGCTGAG
MRP6	AGATGGTGCTTGGATTCGCC	GCCACACAGTAGGATGAATGAG
OCLN	CATTGCCATCTTTGCCTGTG	AGCCATAACCATAGCCATAGC
OSTA	ACCTCGTTTTATGCCGTGTG	AAGAAGGCGTATTGGAAAGGG
OSTB	ATGGTCCTCCTGGGAAGAAGCA	GCCTCATCCAAATGCAGGACTTC
PEPT1	GACAAGCAGTCACCTCAGTAAG	AGTCCCGAGAGCTATCAGGG
PLTP	AAGAGCGGATGGTGTATGTGG	ATGGGGAGTCAATCACTGCTG
SLC9A3R1	GGCTGGCAACGAAAATGAGC	TGTCGCTGTGCAGGTTGAAG
UGT1A1	CTGTCTCTGCCCACTGTATTCT	TCTGTGAAAAGGCAATGAGCAT
UGT1A3	TTTCACCCTGACAACCTATGC	AGCTCCACACAAGACCTATGAT
ZO-1	CAACATACAGTGACGCTTCACA	CACTATTGACGTTTCCCCACTC

1.3 Supplementary Table 3. List of antibodies

Antibodies	Supplier	Host	Dilution
E-Cadherin	Beckton Dickinson	Mouse	1:500
Villin	Santa cruz	Mouse	1:250
TGR5	Abcam	Rabbit	1:200
GLUT2	Santa cruz	Mouse	1:200
Phalloidin 568	Invitrogen		1:500
Alexa Fluor 488	Invitrogen		1:500
Alexa Fluor 647	Invitrogen		1:500
Alexa Fluor 568	Invitrogen		1:500

1.4 Supplementary Table 4. Functions of lipid metabolism markers

Gene	Function
LCT	instructs the production of lactase enzyme(Rings et al., 1994)
SLC9A3R1	encodes the Na^+/H^+ exchanger regulatory factor 1 protein(Lin et al., 2010)
ENPEP	are involved in the control of sodium and water absorption, glucose uptake and absorption and digestion of peptides(Holmes et al., 2017; Penninger et al., 2021)
ACE2	
APOA4	is involved between others in chylomicron assembly, cholesterol transport and blood glucose homeostasis(Kohan et al., 2015)
APOA1	is a major component of the high-density lipoprotein (HDL)(Shioji et al., 2004)
APOA5	is a key regulator of triglyceride levels(Garelnabi et al., 2013)
HMGCS2	encodes the rate-limiting enzyme in the production of ketone bodies(Ruiz-Roso et al., 2020)
PLTP	transfers phospholipid and cholesterol from apo B-containing lipoproteins to HDL(Huuskonen et al., 2001)
ME1	generates nicotinamide adenine dinucleotide phosphate (NADPH) that is used in fatty acid and cholesterol biosynthesis(Jiang et al., 2013)
CYP8B1	is required for the synthesis of cholic acid(Kim et al., 2007)
CLPS	is a cofactor of pancreatic lipase, which allows the lipase to anchor itself to the lipid-water interface(Van Tilbeurgh et al., 1999)
LIPA	produces the lysosomal acid lipase(Zhang, 2018)
LPL	hydrolyses circulating triglycerides and releases fatty acids that can be taken up by tissues(Sylvers-Davie and Davies, 2021)

2 Supplementary Figure

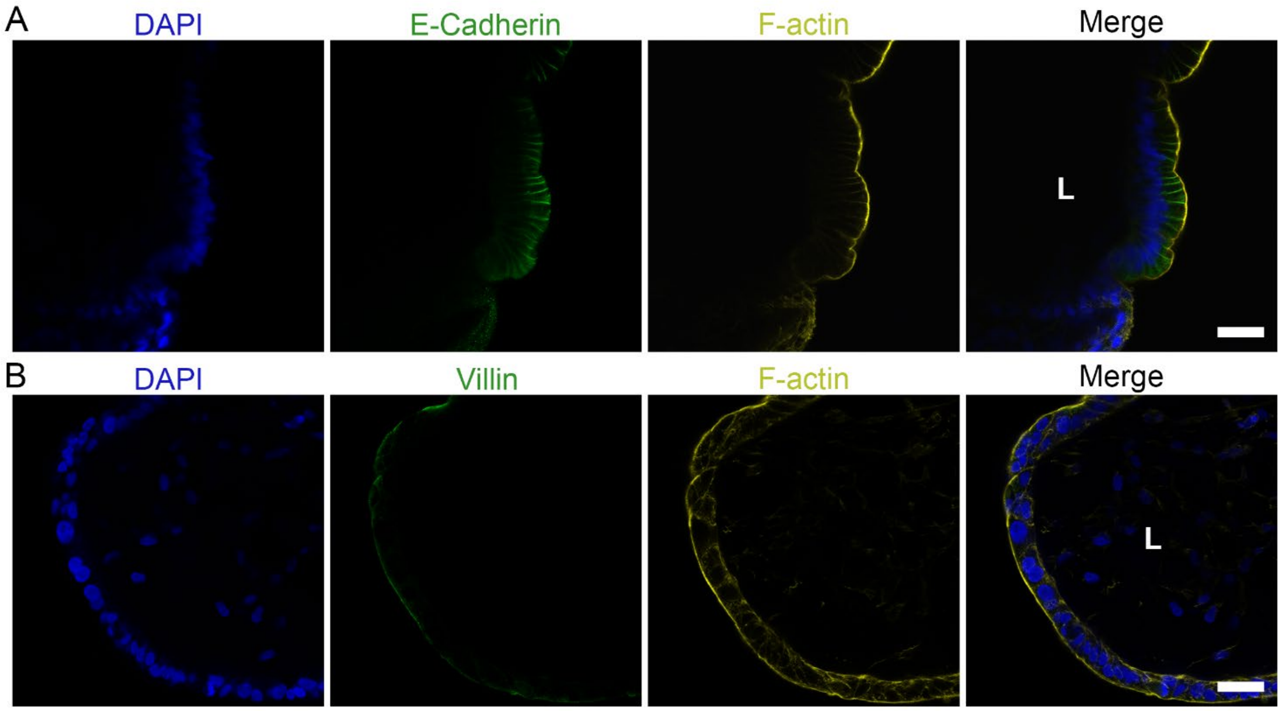

Supplementary Figure 1: Apico-basolateral organization of human intestinal organoids. (A) Immunofluorescence staining for the basolateral marker E-Cadherin (green) and the apical marker F-actin (yellow) indicated reversed polarity, where the apical side is facing outwards and the basal inwards. (B) The apical markers Villin (green) and F-actin (yellow) were co-expressed in the outer surface of the organoids. Scale bars: 50 µm. L: lumen.

3 References

Garelnabi, M., Lor, K., Jin, J., Chai, F., and Santanam, N. (2013). The paradox of ApoA5 modulation of triglycerides: Evidence from clinical and basic research. Clin. Biochem. 46, 12–19. doi:10.1016/J.CLINBIOCHEM.2012.09.007.

Holmes, R. S., Spradling Reeves, K. D., and Cox, L. A. (2017). Mammalian Glutamyl Aminopeptidase Genes (ENPEP) and Proteins: Comparative Studies of a Major Contributor to Arterial Hypertension. J. Data Mining Genomics Proteomics 8. doi:10.4172/2153-0602.1000211.

Huuskonen, J., Olkkonen, V. M., Jauhiainen, M., and Ehnholm, C. (2001). The impact of phospholipid transfer protein (PLTP) on HDL metabolism. Atherosclerosis 155, 269–281. doi:10.1016/S0021-9150(01)00447-6.

Jiang, P., Du, W., Mancuso, A., Wellen, K. E., and Yang, X. (2013). Reciprocal regulation of p53 and malic enzymes modulates metabolism and senescence. Nat. 493, 689–693. doi:10.1038/nature11776.

Kim, I., Ahn, S. H., Inagaki, T., Choi, M., Ito, S., Guo, G. L., et al. (2007). Differential regulation of bile acid homeostasis by the farnesoid X receptor in liver and intestine. J. Lipid Res. 48, 2664–2672. doi:10.1194/JLR.M700330-JLR200.

Kohan, A. B., Wang, F., Lo, C. M., Liu, M., and Tso, P. (2015). ApoA-IV: Current and emerging roles in intestinal lipid metabolism, glucose homeostasis, and satiety. Am. J. Physiol. - Gastrointest. Liver Physiol. 308, G472–G481. doi:10.1152/AJPGI.00098.2014.

Lin, S., Yeruva, S., He, P., Singh, A. K., Zhang, H., Chen, M., et al. (2010). Lysophosphatidic Acid Stimulates the Intestinal Brush Border Na+/H+ Exchanger 3 and Fluid Absorption via LPA5 and NHERF2. Gastroenterology 138, 649–658. doi:10.1053/J.GASTRO.2009.09.055.

Penninger, J. M., Grant, M. B., and Sung, J. J. Y. (2021). The Role of Angiotensin Converting Enzyme 2 in Modulating Gut Microbiota, Intestinal Inflammation, and Coronavirus Infection. Gastroenterology 160, 39–46. doi:10.1053/J.GASTRO.2020.07.067.

Rings, E. H. H. M., van Beers, E. H., Krasinski, S. D., Verhave, M., Montgomery, R. K., Grand, R. J., et al. (1994). Lactase; Origin, gene expression, localization, and function. Nutr. Res. 14, 775–797. doi:10.1016/S0271-5317(05)80212-X.

Ruiz-Roso, M. B., Gil-Zamorano, J., López de las Hazas, M. C., Tomé-Carneiro, J., Crespo, M. C., Latasa, M. J., et al. (2020). Intestinal Lipid Metabolism Genes Regulated by miRNAs. Front. Genet. 11, 707. doi:10.3389/FGENE.2020.00707/BIBTEX.

Shioji, K., Mannami, T., Kokubo, Y., Goto, Y., Nonogi, H., and Iwai, N. (2004). An association analysis between ApoA1 polymorphisms and the high-density lipoprotein (HDL) cholesterol level and myocardial infarction (MI) in Japanese. J. Hum. Genet. 49, 433–439. doi:10.1007/s10038-004-0172-1.

Sylvers-Davie, K. L., and Davies, B. S. J. (2021). Enyzmes | Lipoprotein Lipase. Encycl. Biol. Chem. Third Ed. 3, 307–320. doi:10.1016/B978-0-12-819460-7.00125-0.

Van Tilbeurgh, H., Bezzine, S., Cambillau, C., Verger, R., and Carrière, F. (1999). Colipase: structure and interaction with pancreatic lipase. Biochim. Biophys. Acta - Mol. Cell Biol. Lipids 1441, 173–184. doi:10.1016/S1388-1981(99)00149-3.

Zhang, H. (2018). Lysosomal acid lipase and lipid metabolism: New mechanisms, new questions, and new therapies. Curr. Opin. Lipidol. 29, 218–223. doi:10.1097/MOL.0000000000000507.
